# Risk-based stratified primary care for common musculoskeletal pain presentations: qualitative findings from the STarT MSK cluster randomised controlled trial

**DOI:** 10.1186/s12875-022-01924-3

**Published:** 2022-12-16

**Authors:** Benjamin Saunders, Adrian Chudyk, Joanne Protheroe, Vincent Cooper, Bernadette Bartlam, Hollie Birkinshaw, Nadine E Foster, Jonathan C Hill

**Affiliations:** 1 Primary Care Centre Versus Arthritis, School of Medicine, Keele University, Staffordshire, UK; 2 Faculty of Environmental and Life Sciences (FELS), University of Southampton, Southampton, UK; 3 STARS Research and Education Alliance, Surgical Treatment and Rehabilitation Service, The University of Queensland and Metro North Hospital and Health Service, QLD Herston, Australia

**Keywords:** Musculoskeletal pain, Stratified care, Prognostic risk, Primary care, General practice, Qualitative

## Abstract

**Background:**

The STarT MSK cluster randomised controlled trial (RCT) investigated the clinical- and cost-effectiveness of risk-based stratified primary care versus usual care for patients with back, neck, shoulder, knee or multi-site pain. Trial quantitative results showed risk-based stratified care was not superior to usual care for patients’ clinical outcomes, but the intervention led to some changes in GP clinical decision-making. This paper reports a linked qualitative study exploring how risk-based stratified care was perceived and used in the trial, from the perspectives of clinicians and patients.

**Methods:**

Semi-structured interviews were conducted with 27 patients, and focus groups and interviews with 20 clinicians (GPs and physiotherapists) in the intervention arm of the trial. Data were analysed thematically and findings explored using Normalisation Process Theory (NPT) and the COM-B model.

**Main findings:**

Risk-based stratified care (subgrouping and matching treatments) was found to have ‘coherence’ (i.e. made sense) to several clinicians and patients, in that it was well-integrated in practice, and supported clinical decision-making. However, for some GPs stratified care was less ‘meaningful’, as the risk-stratification tool did not fit with usual ways of consulting and added to already time-pressured consultations. GPs reported giving more patients written information/advice due to easier access to electronic information leaflets through the trial template and were motivated to refer patients to physiotherapy as they believed the trial resulted in faster physiotherapy access (although this was not the case). Patients and clinicians reported that risk-based stratified care influenced conversations in the consultation, prompting greater attention to psychosocial factors, and facilitating negotiation of treatment options. Physiotherapists saw benefits in receiving information about patients’ risk subgroup on referral forms.

**Conclusion:**

These findings provide context for interpreting some of the trial outcomes, particularly in relation to changes in clinical decision-making when risk-based stratified care was used. Findings also indicate potential reasons for lack of GP engagement with risk-based stratified care. Positive outcomes were identified that were not captured in the quantitative data, specifically that risk-based stratified care positively influenced some GP-patient conversations and facilitated negotiation of treatment options.

**Trial registration:**

ISRCTN15366334 (26/04/2016).

**Supplementary Information:**

The online version contains supplementary material available at 10.1186/s12875-022-01924-3.

## Background

Musculoskeletal (MSK) pain is common and has significant impacts for individuals, healthcare and society [1]. In the UK, the majority of MSK problems are managed in primary care settings [2], with 20% of patients per year consulting their general practitioner (GP) with an MSK problem [3, 4]. Usual primary care for MSK pain often follows a ‘stepped’ approach, in which patients are initially offered low intensity treatments (e.g. advice and education, simple pain medications), moving onto more intensive treatments (e.g. courses of physiotherapy) if necessary [5]. An alternative approach to stepped care, is to stratify care according to the patient’s risk of experiencing persistent disabling pain. Risk-based stratified primary care for low back pain (LBP), involving stratifying patients as low, medium or high risk of poor outcome, e.g. persistent disabling pain, and then matching patients to appropriate treatments, has been shown to be both clinically-effective and cost-effective [6–8]. This approach allows patients requiring more intensive treatment to be identified earlier, and to be ‘fast-tracked’ to that treatment. Patients at low risk can be reassured that they have a good prognosis and potentially avoid receiving unnecessary investigations and treatments. Risk-based stratified care, for example using the STarT Back tool [9], is now recommended for LBP internationally in a number of clinical guidelines [10–12].

Building on this evidence, a new risk-stratification tool was developed and validated for use with patients with the five most common MSK pain presentations (the 10-item Keele STarT MSK risk-stratification tool) [13]; and 16 risk-matched treatment options for low, medium and high risk subgroups agreed through stakeholder consensus [14]. Using both the new tool and risk-matched treatment options, this new model of risk-based stratified care was first tested in a feasibility and pilot trial in UK general practice [15, 16] prior to the main STarT MSK trial [17] (Trial registration: ISRCTN15366334; 26/04/2016). The main trial was a two-parallel arm, pragmatic, cluster randomised controlled trial in UK general practices that investigated the clinical- and cost-effectiveness of risk-based stratified primary care versus usual, non-stratified care for patients with back, neck, shoulder, knee or multi-site pain. Twenty-four GP practices were randomised (1:1), in order to recruit 1200 patients. GPs in practices allocated to the intervention arm were supported to deliver risk-based stratified care using a bespoke computer-based template, including the risk-stratification tool, and risk-matched treatment options for patients at low, medium or high risk of poor outcome. The risk-based stratified care model sought to influence GP clinical decision-making to encourage less: prescribing of long-term opioids, neuromodulators, muscle relaxants and corticosteroid injections, referral to imaging and specialist orthopaedics (particularly for low risk patients), and less sick certification; and more: written self-management advice, simple over-the-counter analgesics, early referral to physiotherapy (for medium and high risk patients), plus further GP assessment to address complexities such as comorbidities, distress, and emerging frailty.

The trial results showed no significant difference between intervention and control arms for the primary outcome: time-averaged pain intensity over 6 months. There were no significant differences in most secondary outcomes, including function, MSK health and quality of life, although patients in the intervention arm reported being more satisfied with their care. Through an anonymised medical record audit, significant differences were observed in some aspects of clinical decision-making between stratified care versus usual care; with risk-stratification leading to greater provision of written information, physiotherapy referral, simple analgesics, and short-term prescribing of strong opioids (see [18], for full quantitative trial results).

This paper reports the findings of a linked qualitative study that aimed to understand how risk-based stratified care was perceived and used within routine practice in the trial, from the perspectives of clinicians and patients. This qualitative study was not intended as a formal process evaluation; however, gaining an understanding of how stratified care was perceived and experienced provides important context to support the interpretation of the trial outcomes.

## Theoretical frameworks

Adopting risk-based stratified care in consultations as part of the trial involved GPs changing their clinical behaviour in managing patients with MSK pain. Encouraging clinical behaviour change, particularly within the increasingly high-pressured context of general practice, can be extremely challenging [19]. Examining behaviour change using a theoretically-informed approach can enrich the understanding of identified issues, and, as such, we drew on Normalisation Process Theory (NPT) [20] and the COM-B model [21]. NPT is a framework for exploring the adoption and implementation of healthcare interventions, and can aid the understanding of why some interventions are accepted and more successfully embedded in everyday clinical practice than others [20]. NPT includes four components: coherence (i.e. sense-making); cognitive participation (i.e. engagement); collective action (the ‘work’ done in operationalising the intervention); and reflexive monitoring (how patients, clinicians and other stakeholders appraise the benefits and costs of the intervention). We drew on the ‘coherence’ component, in particular, to inform the interpretation of our findings. Coherence relates to whether, and to what degree, an intervention is perceived to be ‘meaningful’ to key stakeholders, and whether it ‘makes sense’ within the context of a given clinical or healthcare setting. ‘Coherence’ aligns closely with our aims, as the degree to which clinicians and patients perceive risk-based stratified care as ‘making sense’ is key to understanding how it is perceived and used within routine practice.

The COM-B model comprises three key determinants that can inform the understanding of behaviour change: *capability* – the individual’s or group’s psychological or physical ability to enact the given behaviour; *opportunity* – the physical and social environment that enables (or inhibits) the given behaviour; and *motivation* – the reflective and automatic mechanisms that activate or inhibit behaviour. The COM-B is an extension of the Theoretical Domains Framework (TDF) [22]. The TDF combines 112 psychological constructs determining behaviour change into 14 broad domains, which can be used to identify barriers and facilitators to behaviour change in relation to healthcare interventions. The COM-B model synthesises the TDF domains within its three central components. NPT and the COM-B model will inform the interpretation of our qualitative findings, with the aim of developing a robust understanding of the identified issues.

## Methods

One-to-one semi-structured interviews and focus groups were conducted at a single time-point with patients and clinicians in the intervention arm of the main STarT MSK trial. Interviews were conducted with 27 patients; and four focus groups and seven one-to-one interviews were carried out with clinicians (*n =* 20; 13 GPs, 6 physiotherapists, 1 First Contact Practitioner (FCP)^1^). Data were collected between August 2018 and July 2019. Patient interviews were carried out by BS (male, PhD) and HB (female, PhD) (experienced qualitative researchers from a social science and health psychology background, respectively); clinician focus groups were carried out by BS and AC (male, PhD, an academic GP with training in qualitative research), and BS conducted the clinician interviews.

GPs and physiotherapists had differing roles and levels of involvement in the trial. In consultations with eligible patients, GPs and the one FCP in practices allocated to the intervention arm were asked to complete the risk-stratification tool on their computer template and select risk-matched treatment options. One treatment option was referral to physiotherapy, and GPs selecting this option were asked to ensure that the patient’s risk subgroup and risk-stratification tool score (low, medium or high risk) were included in the information on the physiotherapy referral form. Physiotherapists were not involved in risk-stratifying patients, but provided onward treatment for patients who were referred to them from general practice.

The settings were general practices and physiotherapy services in the West Midlands region of England. The study received ethical approval from the NHS REC East Midlands Nottingham 1 (Ref:16/EM/0257).

### Patient and Public Involvement and Engagement

Six patients from the programme’s Patient and Public Involvement and Engagement (PPIE) group provided input into developing the aims and objectives of the qualitative study, as well as giving feedback on qualitative topic guides and patient-facing documentation, through attending an in-person meeting with members of the research team. With regard to the interview topic guides, the PPIE members felt that it was important to explore aspects of the consultation that may be important to patients, but did not directly relate to the use of the risk-stratification tool and matched treatments. This led to the incorporation of questions in the patient interview topic guide on the degree to which patients felt their needs had been addressed by the GP, and whether they felt they had been listened to in consultations.

### Sampling and recruitment

Patients were recruited by invitation letter and then via phone, having consented to further contact in the first questionnaire completed as part of the trial. Thirty-seven patients who were sent an interview invitation letter did not respond to the invitation by returning the freepost reply slip that was included, so were not contacted via follow-up telephone call. Patients were purposively sampled to capture diverse characteristics including MSK pain site, risk group allocation, geographical area, and participant demographics: e.g. age, gender, occupation, ethnicity.

Clinicians agreed to participate in focus groups as part of their participation in the trial. Clinician focus groups were arranged in conjunction with trial feedback sessions, which were held at intervention practices around six weeks after the start of the trial in their practice (see [23], for details of clinician feedback during the trial). Focus groups were conducted at four GP practices following these feedback sessions. The four GP practices were sampled for variation in practice size, geographical location, and sociodemographic composition of their patient population. Physiotherapists from services linked to these practices were contacted via email to invite them to participate in a focus group.

In order to access a broader range of experiences, we also invited clinicians from other intervention practices to take part in one-to-one telephone interviews, to supplement the focus group data. Clinicians were initially approached via email (in the case of some GPs via their practice manager), followed by a telephone call or email to confirm interview arrangements. Clinicians invited for interview were sampled for variation in geographical location. We also aimed to sample GPs for variation in levels of engagement with the intervention; i.e. those who had completed the risk-stratification tool and selected appropriate risk-matched treatments for a high proportion of eligible patients, as well as those who had engaged less. However, this proved challenging, as those GPs who had not strongly engaged with the intervention were also less willing to take part in the qualitative study; therefore, those who took part had generally shown higher levels of engagement with stratified care. Eleven GPs who were invited for interview either declined citing lack of time to participate, or did not respond to the invitation.

### Data collection

Of the 27 patient interviews, 18 took place at participants’ homes; and nine via telephone, in line with participants’ preferences. Interviews lasted between 37 min and 1 h 23 min. Clinician focus groups lasted between 49 min and 1 h 17 min; telephone interviews with clinicians lasted between 26 and 57 min.

Interviews and focus groups were audio-recorded. All participants were given an information letter explaining the study prior to providing written informed consent at the start of interviews/focus groups, or audio-recorded consent in the case of telephone interviews. Field notes were not made during interviews or focus groups or as it was felt this could negatively impact upon the rapport between researcher and participants. Consent was reaffirmed verbally at the end of each interview/focus group.

Separate topic guides were used for patient and clinician interviews/focus groups. Initial topic guides were developed based on the trial aims and outcomes of interest, as well as covering a range of areas relevant to the qualitative study aims. These initial topic guides began with a small number of broad questions, and were iteratively revised and added to throughout the data collection process, based on emergent findings. The final versions of the topic guides are displayed in Supplementary files 1 and 2. A semi-structured approach was adopted during interviews; therefore, topic guides were not followed rigidly, and the researchers retained flexibility to follow up on any unexpected findings emerging during the interview/focus group.

### Data analysis

Data analysis took place before the results of the trial were known; therefore, analysis was inductive, and was not geared towards trying to explain specific trial outcomes. However, where the trial quantitative results are able to be illuminated by the qualitative data, this is provided.

Audio-recordings of interviews and focus groups were transcribed and anonymised. A two-stage analysis framework was adopted; first, an inductive thematic analysis, followed by mapping the identified themes onto the two theoretical frameworks: the NPT ‘coherence’ construct and three COM-B determinants. Analysis was an iterative process and data collection continued until saturation was judged to have been reached, defined as ‘informational redundancy’‒ the point at which additional data no longer offers new insights [24].

Anonymised transcripts were first systematically coded on a line-by-line basis by BS, with the aid of the software program Nvivo 12, in order to identify recurrent concepts inductively. Coding was at first largely descriptive, and later became more conceptual as interpretations of the data moved towards a higher level of theoretical abstraction. Coding was reflexive and recursive, with codes being revisited in light of the findings of subsequent data-collection. Analysis began with the patient data and then mapped the views/experiences of clinicians against that, to allow for direct comparison between the two. Member-checking, that is, sending transcripts and findings to participants for comment and feedback, was not used. This was because analysis was primarily across-case, rather than within-case, which meant it would have been difficult for participants to feed back on the validity of the interpretations of their own data when included within the broader analysis of the dataset as a whole.

Data analysis was discussed at regular meetings between team members from different disciplinary backgrounds (BS: social science; HB: health psychology; JH: physiotherapy; VC, JP, AC: general practice), allowing for cross-disciplinary perspectives on the data, leading to the development of three main themes. Themes were then explored in relation to how well they ‘fitted’ [25] within the parameters of the NPT and COM-B components. We explored the degree to which the identified themes could be seen to ‘fit’ within these frameworks, and how the theoretical constructs manifested in relation to these themes.

In what follows we outline the characteristics of the participant sample, before reporting the key themes.

## Results

### Patient participant characteristics

Of the 27 patients that were interviewed, 16 identified as female and 11 male, aged from 19 to 84 years (average age: 63). Fourteen participants were retired and eleven were in paid employment, representing a range of occupation types. One patient was not in paid employment and one did not report their employment status. Six patients had presented to the GP with shoulder pain, eight with knee pain, eight with back pain, three with multisite pain and two neck pain. At the point-of-consultation, three patients were allocated to the low risk subgroup, seventeen to medium risk, and seven high risk. Patients came from each of the four different geographical areas in the trial: Staffordshire, Shropshire, Warwickshire, Cheshire. Whilst a varied sample was achieved in relation to the above characteristics, we were not able to achieve a varied sample with regards to ethnicity; 25 patients self-reported as white British, 1 patient as Asian and 1 patient as being of mixed ethnicity. Table 1, below, summarises the characteristics of the 27 patients interviewed:

**Table 1:** Characteristics of patients interviewed

Patient ID	Gender	Age	Pain site	Risk subgroup	Occupation type	Ethnicity
1	Male	43	Shoulder	Medium	Managerial	Asian
2	Male	36	Shoulder	Medium	Managerial	Mixed
3	Male	49	Knee	Medium	Managerial	White
4	Male	50	Back	High	Manual	White
5	Female	59	Shoulder	Medium	Retired	White
6	Female	19	Knee	Medium	Service	White
7	Female	67	Shoulder	Medium	Retired	White
8	Male	71	Knee	High	Retired	White
9	Male	67	Multisite	High	Retired	White
10	Female	62	Back	High	Unreported	White
11	Female	58	Back	Medium	Service	White
12	Female	58	Neck	Medium	Service	White
13	Female	51	Knee	High	Service	White
14	Female	40	Knee	Medium	Service	White
15	Female	59	Knee	Low	Self-employed	White
16	Male	82	Shoulder	Low	Retired	White
17	Male	69	Back	Medium	Service	White
18	Male	81	Knee	Medium	Retired	White
19	Female	80	Neck	Medium	Retired	White
20	Female	73	Shoulder	Medium	Homemaker	White
21	Male	67	Back	Low	Retired	White
22	Female	70	Back	Medium	Retired	White
23	Male	83	Back	High	Retired	White
24	Female	67	Multisite	High	Retired	White
25	Female	75	Knee	Medium	Retired	White
26	Female	84	Back	Medium	Retired	White
27	Female	79	Multisite	Medium	Retired	White

### Clinician participant characteristics

Twenty clinicians took part in a focus group or interview. Thirteen clinicians identified as female and seven male. Of these, seven GPs were female and six male, five physiotherapists were female and one male, and the one FCP was female. The length of time clinicians had been practising ranged from newly qualified to over 25 years in practice. Clinicians were reasonably equally spread across the four geographical regions involved in the trial. On average, the 13 GPs and 1 FCP completed the trial recruitment template in 39% of eligible patients. Across all participating GPs in intervention practices in the trial, the risk stratification tool was completed in 30% of eligible patients; therefore, those who participated in the qualitative study had generally slightly higher engagement with risk-based stratified care.

### Main themes

The three themes identified were:

1. Perceived influence of risk-based stratified care on clinician-patient communication.
2. The role of risk-based stratified care in informing clinical decision-making and its ‘fit’ within consultations.
3. Implications of risk-based stratified care for inter-professional working.

#### Theme 1: Perceived influence of risk-based stratified care on clinician-patient communication

Many GPs reported that the use of stratified care influenced the conversations they had with patients. For instance, the approach prompted them to better explore psychosocial issues related to the MSK pain presentation. This was particularly the case for shoulder and knee pain presentations, where they reported previously having taken a more biomedical approach and less commonly explored the broader impact of pain on the patient:

*For the vast majority of people there’s definitely value added [in the stratified care approach] because you’re actually putting [the pain] much more in context for that individual rather than “oh you’ve got adhesive capsulitis [i.e. frozen shoulder], this is what happens with adhesive capsulitis, do you want your injection now or later?”. Whereas actually understanding how that affects them…uncovering much more psychological issues with anxiety, that you perhaps otherwise wouldn’t have got on to. (Male GP 3, focus group 1)*

Patients’ experiences complemented this finding as patients highlighted added value from GPs discussing the psychosocial impacts of their pain during the consultation. In particular, they reported that risk-stratification tool items that focused on mood facilitated a more holistic approach, allowing them the ‘opportunity’ to talk about broader issues that may be underlying their pain:

*I: do you feel [the questions] add much to the consultation?*

*P: Yes, I do because I think…you can often go to the doctors presenting with one thing but there could be an underlying…more emotional, psychological based problem. So I do think it’s good to be asked those sorts of things and given an opportunity to talk about the wider ‘you’ rather than just the one bit that happens to be hurting at the moment. (Female patient, 59, medium risk, shoulder pain)*

Some patients reported that certain risk-matched treatments they had received, such as social prescribing, differed from those they would usually associate with GP management options, and were surprised to be offered these:

*I just never associated Slimming World with a sort of thing a doctor would suggest you do, but it was obviously good advice…suppose psychologically I can see things are better than they were. I get upstairs relatively easily now which I would always hope to be able to it was just a bit worrying when I found I couldn’t.*

*(Male patient, 81, medium risk, knee pain)*

GPs highlighted the positive influence of the risk-based stratified approach on difficult conversations with some patients who have expectations of an imaging referral where there may not be a clinical need. The GPs reported using the risk-matched treatment options to support them in having these conversations, through facilitating negotiation with patients, between the risk-matched treatments and the patients’ expectations; for instance, as a way of convincing patients that a scan may not be the most appropriate option:

*[Stratified care] probably concentrates or focuses decision making in that sense rather than making it truly shared, because if patients come in with a perception about investigation and referral and we’re deflecting them from that to something else then it’s more of a negotiation than a shared decision making− “you come in with this perspective, I’ve got this perspective, where can we find some middle ground”. (Female GP 1, focus group 2)*

The GP in the above extract talked of ‘deflecting’ the patient away from discussion of potential investigations, which may suggest that decision-making was steered by the GP. However, the GP indicates that having convinced the patient that a scan may be unnecessary, they will engage in a negotiation about management options. Whilst the GP perceived ‘negotiation’ as different to their understanding of shared decision-making, it could be suggested that this negotiation indicates a form of shared decision making, that is, reaching agreement through finding ‘some middle-ground’. GPs reported that one of the reasons risk-based stratified care was successful in facilitating this negotiation was that patients had confidence in the use of an evidence-based approach:

*You may get somebody who’s very fixated on what they want out of [the consultation], but I think the tool is quite useful in general because I find that when people can sort of see it and feel that it’s evidence-based and that it’s taking them in a safe direction then I find generally most people are happy with that. (Male GP 1, focus group 4)*

However, whilst some GPs reported using risk-based stratified care to support conversations about management options and manage patient expectations, most patients reported being unaware of the role of the risk-stratification tool in informing management. This suggests that either this was not communicated to them by their GP, or they did not recall this being explained to them in the consultation. In some cases, patients perceived the tool questions as providing information to inform a broader understanding of MSK pain management as part of the research, rather than primarily supporting their own care:

*From what I can remember, I think he did sort of vaguely explain that it might help me, but this was looking at a wider picture about how pain and pain management is dealt with by GPs and wider professionals.*

*(Female patient, 67, high risk, multisite pain)*

#### Theme 2: The role of risk-based stratified care in informing clinical decision-making and its ‘fit’ within consultations

There was some variation in clinicians’ views regarding the role of risk-based stratified care in informing their treatment decision-making. Some GPs, and the one FCP, reported that the risk-matched treatment options were of use, either in confirming and validating their decision-making, or in some cases directing their decisions:

*[The risk-matched treatments] often confirm the decision I’d already made. But a couple I have been surprised at. The majority I agreed with. I would say it’s working because there’s a couple of them that I potentially wouldn’t have sent to physio as early as the tool had then made me send them. So yeah, some of them I would’ve probably kept in my little hands, and said ‘Right, go and try this and if that doesn’t work, then refer yourself to physio’ (Female FCP, clinician interview 1)*

However, some GPs felt that the risk-matched treatments had less influence on management; they reported preferring to make decisions based on their own clinical judgement, indicating a lack of ‘buy in’ to the stratified care approach:

*I don’t think I’m letting the template make the decision. I’m just doing what I would normally do and then if it’s fitting, great but if it’s not, I’m probably still doing it…so I don’t think it’s made a substantial difference. (Female GP, clinician interview 2)*

Other GPs felt that the risk-stratification tool and matched treatments did not influence their clinical decision-making because they experienced difficulties in fitting the approach into their usual way of consulting. The trial recruitment template activated when GPs entered a symptom or diagnostic code, and they reported that because they usually enter this code at the end of the consultation, this meant the template activated once they had already reached a decision on management:

*[The trial recruitment template] tends to be coming in at the end. Maybe if I fired it off at the beginning. If I wrote the problem title at the very beginning as the patient was in the room, it may affect what I’m doing but normally, you’ve already had the consultation and you’ve already had that discussion with the patient. You’re then writing up your notes towards the end and then that’s when it comes up. (Male GP, clinician interview 5)*

Some GPs highlighted the time that it took to complete the tool and risk-matched treatments within the consultation as being a barrier to stratified care changing their clinical behaviour. This was seen as problematic particularly when MSK pain was only one of several problems the patient was presenting with, which led to a desire to escape from the trial recruitment template:

*If you get it as one of a multiple number of problems in the consultation, it’s hard work…it certainly takes you more time. So you get this sigh when you put in a read code and it flashes up with the study. You have to think of something else and you can’t get out, the only way you can get out of it is to end a consultation and put in a different read code. So it doesn’t fit in very well and I don’t think it changes what I do, I don’t think it really makes a lot of difference to that to be honest. (Female GP, focus group 2)*

However, several GPs discussed changes to their clinical behaviour as a result of using risk-based stratified care. Whilst there was little discussion of changes in prescribing over-the-counter medications or strong opioids (which was shown in the trial quantitative results), a number of GPs reported that they provided patients with written information more commonly than they had previously, due to these resources being easily accessible electronically via the template, which they viewed very positively:

*I just love the fact that you can just click on it and there’s the exercise sheet at the click of a button, that’s fantastic…and certainly I find it useful. As a GP I know all those leaflets are there, but linking them so that you can print them off straight away is quite a good thing. You know, you can fill my drawer forever with a set of leaflets, but I’ll never use them. (Female GP, clinician interview 4)*

Patients reported finding this written advice straightforward and easy to follow:

*P: He gave me a leaflet with some basic exercises, just gentle things that I could do at home while I was sitting or standing. Just to keep mobile.*

*I: And have you been able to follow those, have you been able to do them at home?*

*P yes, I have, yes. (male patient, 67, low risk, back pain)*

A number of GPs reported that their decision to refer patients at medium or high risk to physiotherapy was driven by their perception that these patients would be seen more quickly as part of the trial, despite this not being the case.

*I think the big advantage really is if they’re scoring medium or high we know we’ve got a trial physio to pick them up and I can talk about that…I’m happy to use a trial referral because they’re quicker. (Male GP, clinician interview 3)*

Patients who consulted with a physiotherapist also considered their waiting time to have been shorter than it would have been as part of usual care, and some reported that this had influenced their view towards being referred:

*I think it [receiving physiotherapy] was quicker than usual. Again, that was one of the reasons why it was felt that it was the right thing to do because I think [the GP] said it was a fast-track process to get the physio. I think normally, you have to wait 12 weeks but I think because I was taking part in the study, it was a lot shorter. I think it was about four weeks. (Male patient, 43, medium risk, shoulder pain)*

#### Theme 3: implications of risk-based stratified care for inter-professional working

Whilst not a principal objective of the intervention, it was hoped that the use of risk-based stratified care might lead to improved communication between GPs and physiotherapists, particularly in relation to the management of complex patients. With regard to information-sharing, physiotherapists indicated having received more information from GPs in intervention practices than they would with usual patient referrals, and they reported finding additional risk group information useful in alerting them to areas they might need to explore, particularly with patients at high risk:

*It just gives me a little bit of a better idea of how much more of a discussion is needed, how much more of an education is needed as opposed to just the physio intervention. If someone’s got those higher risk factors, it might sway me into more of the discussion, seeing…how happy patients are with their condition, how much information you think they need or how much education they might need. Perhaps there’s a bit more of a focus on that with those higher risk ones…it gives me a heads-up with where to start with someone. (Female physiotherapist, clinician interview 6)*

However, some physiotherapists noted that patients often presented differently than was expected from their risk-stratification subgroup or tool score from the GP. This information was felt to be useful as an indication of whether the patient had improved since their GP consultation. However, they reported that their management was driven by how the patient presented at the time (typically 6–8 weeks later) rather than relying on the risk group allocation from the GP:

*I take more of what the patient presents now; more so than sticking to those categories. If someone might present on paper as a high risk or a medium risk but then in person present differently, I then would probably change my plan…I’ll be more guided by the patient on how they present at that first appointment. (Female physiotherapist, clinician interview 7)*

Patients highlighted the importance they placed on healthcare professionals such as GPs and physiotherapists communicating with each other about their care:

*[The physiotherapist] asked me what the GP had said. And I said, he said he was probably going to refer me to the surgeon and maybe for this special…steroid injection… She’s going to ring my GP she said. Have a long talk with him about the possible treatment and then somebody will get back to me. So I was quite pleased that she wanted to talk to the GP about me. (Female patient, 67, medium risk, shoulder pain)*

However, clinicians did not feel that being part of the trial had resulted in them more regularly discussing complex patients. In some cases, clinicians felt that close GP-physiotherapist communication was already well-established:

*I’ve not noticed anything different to what we used to have. Certainly we already had a very good working relationship. The physios there used to contact us anyway. So that had been established. But certainly I’ve had no more contact than we would’ve had prior to the study. (Female GP, clinician interview 4)*

Whilst the use of risk-based stratified care did not result in closer inter-professional communication, some physiotherapists reported positive views about having a dedicated email address as part of the intervention for contacting GPs in intervention practices, despite not having needed to make use of it up to that point:

*I think the comfort for us…it’s just that we know that because if we do get these patients and we do have an issue, we know we can come back to you quite easily, because it’s all part of the trial. I think that’s probably the main thing from our point of view. Like I said, I’ve not felt I’ve needed to as yet. (Male physiotherapist, focus group 3)*

**Fig. 1:**
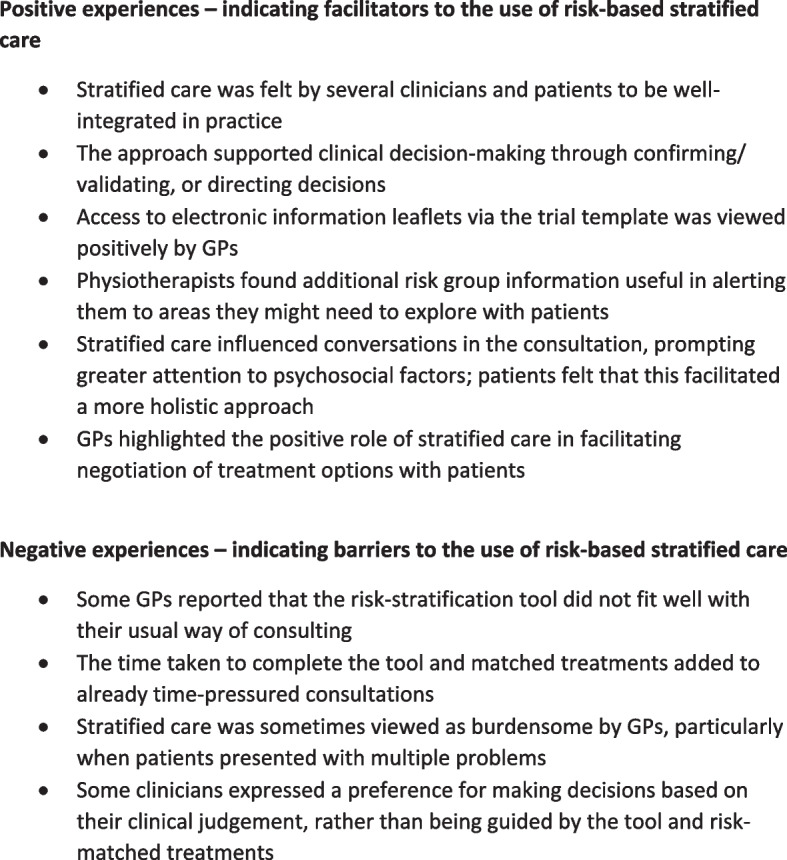
Key positive and negative experiences of risk-based stratified care

Drawing together the findings presented above in relation to each of the three themes, we can identify key positive and negative experiences of risk-based stratified care that run throughout the findings. These are summarised in Fig. 1, below:

## Discussion

### Exploring identified themes in relation to normalisation process theory (NPT) and the COM-B model

In relation to the first identified theme, both patients and clinicians saw added value in the risk-stratification tool and matched treatments in facilitating them in opening up conversations about psychosocial issues related to the patient’s MSK pain. When explored through the lens of NPT, stratified care could be seen to have strong ‘coherence’ (i.e. made sense) in this regard. To establish coherence, an intervention must not only fit well with existing ways of working, but must be ‘distinguishable’ [26] from usual practice so that the purpose of the intervention and its potential benefits are clearly understood [27]. Several clinicians were able to distinguish risk-based stratified care from usual, non-stratified care for MSK pain presentations, in terms of its focus on prognostic risk, which prompted them to explore psychosocial issues underlying the patient’s pain, whereas they felt that usual care more often adopted a biomedical focus. They also saw a ‘clear purpose’ with regard to the added value that risk-matched treatment options had in facilitating difficult conversations with patients.

Patients were unaware that risk-based stratified care was being used by their GP, and findings suggest that either GPs did not explain to them the relevance or use of the risk-stratification tool to inform management, or that patients did not recall this being communicated to them. However, when this was highlighted to them in interviews, patients reported that tool items facilitated a more holistic approach, which they responded to positively, therefore the approach was seen to ‘make sense’ to them. For patients, exploring the psychosocial impact of pain was seen to open up a broader conversation with the GP; therefore, in relation to the COM-B model (Capability, Opportunity and Motivation), the COM-B determinant of Opportunity is relevant here. However, Opportunity related not only to the opportunity to engage with the intervention, but also the additional opportunities patients and GPs perceived that risk-based stratified care facilitated within the consultation.

In relation to the second identified theme, for some GPs, stratified care was seen to influence their clinical decision making, in terms of either confirming and validating their decision-making, or in some cases directing their decisions. This again demonstrates ‘coherence’ in terms of the benefits of the approach being recognised and understood. In relation to the COM-B model, this evidence of ‘coherence’ appeared to indicate that several of the clinicians interviewed saw themselves as having both the relevant capabilities and available opportunities to successfully deliver stratified care. However, for some GPs stratified care lacked coherence, as their usual way of consulting included inputting the symptom or diagnostic code into the GP computer system towards the end of the consultation. This meant that the electronic pop-up computer prompt to use the trial recruitment template activated later in the consultation, making it less ‘meaningful’, as its influence on treatment decision-making was reduced. They therefore did not feel they had the *opportunity* to fully engage with the intervention. For some GPs, they felt that the risk-stratification tool and risk-matched treatments did not influence their clinical decision-making because they had a preference for relying on their own clinical judgement, and therefore did not identify the approach as having added value. In relation to the COM-B determinant of Motivation, the degree of added value that stratified care was perceived as having was a key motivator for engagement. The additional time taken within the consultation to complete the tool was also seen to be burdensome to some GPs, particularly when patients presented with multiple problems, further impacting their *motivation* to use stratified care.

With regard to the third identified theme, it was found that stratified care did not result in closer inter-professional communication about complex patients, despite patients and clinicians reporting positive views about closer communication between GPs and physiotherapists. In relation to the COM-B model, there appeared to be a lack of *motivation* on the part of clinicians to communicate more closely with each other about patients as a result of using the new approach. This could be because, as reported earlier, there was already good communication between GPs and physiotherapists in some practices, therefore there was a lack of coherence in terms of the approach being ‘distinguishable’ from usual practice in encouraging inter-professional communication.

### Implications of findings for supporting the interpretation trial outcomes

The trial results showed that GPs in stratified care practices completed the risk stratification tool in 1056 (30%) of 3548 possible consultations. Whilst the GPs who participated in the qualitative study had, on average, higher engagement (see Clinician Participant Characteristics, earlier), some findings provide possible explanations for understanding why GPs often did not use risk-based stratified care. In particular, the finding that some GPs experienced difficulties integrating stratified care within their usual way of consulting, and found the time taken to complete the tool and risk-matched treatments burdensome when patients presented with multiple problems, may have been barriers to using the approach. Additionally, the broader challenges of engaging GPs in research, given the increasing demands on their role, should be acknowledged [28]. This may be an even greater barrier in the rapidly changing landscape of primary care post-COVID 19, which could point to the need for further research exploring ways to increase GPs’ motivation to engage in healthcare research.

In the trial quantitative results, when risk-based stratified care was used, GPs reported selecting an appropriate risk-matched treatment option in over three-quarters of patients. This good level of engagement with the risk-matched treatment options could be partly explained by the added value highlighted by several clinicians, in that risk-matched treatments were used to confirm and validate their clinical judgement, direct their management decisions in some cases, as well as facilitate discussions around management with patients. However, there was also evidence that some GPs preferred to rely on their own clinical judgement in cases where this did not correlate with the risk-matched treatments. These GPs did not appear to see added value in the risk-matched treatments, and this lack of ‘buy in’ could indicate one reason why in the remaining quarter of consultations in intervention practices, GPs did not select an appropriate risk-matched treatment option.

The trial results showed no significant difference between intervention and control arms for the primary outcome: time-averaged pain intensity over 6 months, and no significant differences in most secondary clinical outcomes. Whilst these results suggest that this model of risk-based stratified primary care did not lead to improved patient outcomes for patients with MSK pain, the qualitative findings suggest positive outcomes that were not captured in the quantitative data, in particular around the therapeutic alliance. Both patients and GPs who took part in the interviews reported that stratified care led to improved communication, through prompting GPs to explore psychosocial issues underlying the patient’s pain, and facilitating negotiation of differing expectations/priorities. Physiotherapists also highlighted benefits in relation to the additional information they received with GP referrals.

In the trial the anonymised medical record audit demonstrated that risk-based stratified care led to some significant changes in clinical decision-making compared with usual care, including increases in the provision of written information and physiotherapy referral. GPs in the qualitative study reported that having easier access to printable information leaflets and resources via the trial template encouraged them to provide these resources to patients more frequently, which may help to explain this increase in the provision of written information. More referrals to physiotherapy for medium and high risk patients was an intended outcome of the risk-based stratified care model; however, increases were also seen for the low risk subgroup despite this not being a risk-matched treatment option. It may be that the belief held by many patients and GPs that patients received faster physiotherapy access as part of the trial, can help to explain this increase in physiotherapy referral. Given that physiotherapy waiting times were not altered in the trial, this could reflect that this aspect of the trial was not effectively communicated by the trial team to participating GPs. Risk-based stratified care also led to an increase in the provision of simple over-the-counter analgesics and prescription of short-term courses of strong opioids; however, there were no qualitative data that can help to explain these changes.

### Comparison with previous research literature

Our findings show similarities and differences from our own previous qualitative research with patients and clinicians carried out as part of the STarT MSK feasibility and pilot trial [16]. In line with these findings, several GPs and patients who were interviewed saw the risk-stratification tool as a positive addition to consultations, particularly in terms of prompting GPs to explore psychosocial issues that may be underlying the patient’s pain. However, in the pilot and feasibility trial, the wording of some of the tool items was reported by both patients and GPs to be ‘cumbersome’ and as not fitting well within the conversation in consultations. This issue was not reported in this study, which may reflect the success of the amendments that were made to the intervention ahead of the main trial based on these pilot and feasibility trial findings [15, 16].

Similar to our earlier paper [16], many clinicians who were interviewed reported seeing added value in the use of the risk-matched treatment options in informing treatment decision-making. However, there was some variation, with some GPs reporting that the risk-matched treatments did not have a big impact on their clinical decisions, indicating a lack of ‘buy-in’ to the approach. This shows some similarity with Hsu et al’s [29] US-based implementation study using STarT Back; they too found that some primary care professionals expressed a preference for making treatment decisions based on their own clinical judgement rather than being directed by a subgrouping tool.

Qualitative research into the views of patients and clinicians on the use of risk-based stratified care for LBP in Germany [30, 31] found that patients were not convinced about the idea of being matched to treatments based on their risk of poor outcome and placed greater importance on receiving a diagnosis for their pain condition. Whilst GPs in the present study reported that patients presenting with MSK pain commonly had the expectation of being referred for imaging in order to identify the underlying cause of their pain, they were able to manage these expectations through using stratified care to facilitate negotiation between the risk-matched treatment options and patients’ preferences. Karstens et al. [30] also reported that GPs felt there was the potential for stratified care to negatively impact upon the therapeutic relationship through undermining GP-patient rapport; however, this was not a concern raised amongst our participants. Our findings again show more similarity with Hsu et al’s [29] US-based study using STarT Back; they too reported that primary care clinicians were able to use stratified care to facilitate conversations with patients about their concerns.

There were some similarities with Sanders et al’s [32] findings as part of an implementation study of STarT Back (IMPaCT Back) [7]; GPs reported difficulties integrating the STarT Back tool and matched treatments into routine consultations, which was a barrier to its use. However, our findings were more positive; whilst some GPs reported difficulties incorporating risk-based stratified care into their usual way of consulting, others reported that the approach fitted well within the consultation, and saw added value in the tool and risk-matched treatments.

Outside of the stratified care literature, recent studies have identified barriers to addressing psychosocial issues in primary care consultations with patients with MSK pain. A recent systematic review of 25 qualitative studies identified that whilst primary care professionals generally demonstrated an awareness of the impact of psychosocial issues in contributing to experiences of MSK pain, they experienced difficulties in identifying and managing these issues, as well difficulty in ‘apply[ing] the biopsychosocial model holistically’ [33]. Similarly, in a UK-based focus group study with primary care professionals and patients, Gordon et al. [34] found that patients often felt unsupported by professionals in relation to managing the emotional impact of pain, although they focused only on people with chronic pain problems. These findings differ from our study, as it was reported that the use of risk-based stratified care aided GPs in exploring the psychosocial issues underlying the patient’s pain − particularly in the case of shoulder and knee pain. This may suggest that the use of the STarT MSK risk-stratification tool and risk-matched treatment options can help to address some of the barriers to identifying and managing psychosocial issues for MSK pain identified elsewhere in the literature.

### Strengths and limitations

A strength of this study is the parallel investigation of the views of patients, GPs and physiotherapists, allowing access to a range of different perspectives on the use of risk-based stratified care in the trial. The multidisciplinary team involved in data analysis was also a strength, which increases the trustworthiness of the findings presented. Additionally, the use of the two theoretical frameworks─ NPT and COM-B─ enabled us to develop a more robust understanding of the identified themes.

The GPs who were interviewed completed the trial recruitment template with 39% of eligible patients, on average. Whilst this is slightly higher than the average of 30% in stratified care practices across the trial, this may be seen as a strength of the qualitative study, as it does not appear that there was selection bias whereby only those GPs who had high engagement with the intervention agreed to be interviewed, i.e. those who used the stratification tool with the majority of eligible patients. This meant that we were able to access a range of perspectives on the STarT MSK intervention, both positive and negative, and indeed the findings presented indicate some potential reasons for lack of GP engagement in the trial.

A potential limitation is that in some cases patients and GPs were interviewed several weeks after the consultation, which may have hampered recall. Another limitation is that, despite hoping to recruit a sample of patients from diverse ethnic backgrounds, all participants except two were white British; therefore, we are very limited in our understanding of the views of people of different ethnicities towards risk-based stratified care. This lack of ethnic diversity mirrored overall recruitment to the trial. Recruitment of GP practices to participate in the trial relied on willing practices that had the capacity to deliver the intervention. In addition, the tools were all English language, which ruled out most inner city, deprived and ethnically diverse practices, a weakness of many studies in primary care.

One aspect that could be viewed as both a strength and limitation is the timing of data analysis. Data were analysed prior to knowing the trial results. This is a limitation in that it meant that it was not possible to focus the analysis on trying to explain the trial results. However, as previously mentioned the nested qualitative research was not intended as a formal process evaluation: analysing the data prior to knowing the trial results allowed for an inductive exploration of patients’ and clinicians’ perspective that was not biased by looking for particular findings within the data.

When interpreting the findings, it is important to acknowledge the influence of the researchers on participants’ responses in focus groups and interviews, and the influence of the research team’s respective backgrounds on the analysis of the data. The research team’s close involvement with the trial could have influenced the data generated and the data analysis. However, a reflexive approach was adopted throughout, in which the researchers attended to, and acknowledged any biases and preconceptions, through regular reflexive group discussions. In particular, some members of the team involved in the analysis worked either currently or previously as GPs (JP, VC, AC), and these clinical backgrounds will likely have influenced their interpretations of the data. This may have resulted in preconceptions about primary care management of MSK pain that were informed by their own management in consultations; however, this may also have been of benefit in interpreting the GP participants’ reported views and experiences. Patients and clinicians were made aware that the researchers conducting the focus groups and interviews were part of the study team that was testing risk-based stratified care; however, it was also explained to participants that the team were interested in understanding both positive and negative aspects of participants’ experiences.

## Conclusion

This paper reported the views of patients and clinicians towards risk-based stratified care for the most common MSK pain presentations in primary care, tested as part of the STarT MSK trial. The findings provide context to support the interpretation of some of the trial outcomes, particularly in relation to GP engagement with risk-matched treatment options and the changes observed in some aspects of clinical decision-making. Findings also indicate potential reasons for lack of GP engagement with risk-based stratified care. Some positive outcomes in the use of stratified care were also identified that were not captured in the quantitative data. GPs and patients both reported that stratified care positively influenced conversations within the consultation, enabling a more ‘holistic’ focus through paying greater attention to the psychosocial aspects of pain, and facilitating negotiation of treatment options, leading to improved shared decision-making. These findings are important particularly in the context of recent UK Medical Research Council guidance [35], which emphasises that evaluating complex interventions goes beyond asking ‘whether an intervention works in the sense of achieving its intended outcome’ and should consider the broader range of impacts of the intervention within the clinical context in which it is used. The findings presented in this paper indicate that, whilst the risk-based stratified care model tested did not lead to superior clinical outcomes than usual primary care for MSK pain, it was felt by clinicians and patients who took part in interviews to enhance some aspects of primary care consultations.

## Supplementary Information

**Additional file 1.** Focus Group/Interview Topic Guide: Healthcare Professionals.

**Additional file 2.** Interview Topic Guide: Patients.

## Data Availability

In line with the Standard Operating Procedures in place at Keele School of Medicine, where this study was conducted, data are archived at a dedicated location within the Keele University’s network. A request to access archived data can be made by completion of a Data Transfer Request form, which can be accessed by contacting: Primary Care Centre Versus Arthritis, School of Medicine, Keele University, Staffordshire, ST5 5BG, UK; Tel: +44 (0) 1782 733,905.

## Abbreviations

GP: General Practitioner/ General Practice
RCT: Randomised Controlled Trial
COM-B: Capability, Opportunity, Motivation and Behaviour
NPT: Normalisation Process Theory
NHS: National Health Service

## Footnotes

1 In the UK, First Contact Practitioners (FCPs) are utilised in primary care as the first point-of-contact for MSK presentations, to provide early, specialist assessment without the need for a GP consultation. The FCP role is typically carried out by advanced practice physiotherapists working within general practices.
