# Supplementary material for: Risk-based stratified primary care for common musculoskeletal pain presentations: qualitative findings from the STarT MSK cluster randomised controlled trial
Source: BMC Prim Care. 2022 Dec 16;23:326. doi: 10.1186/s12875-022-01924-3 (PMC9754991; doi:10.1186/s12875-022-01924-3)
Supplement: Supplementary file 1 — Additional file 1. Focus Group/Interview Topic Guide: Healthcare Professionals. [file 12875_2022_1924_MOESM1_ESM.docx]

Focus Group/Interview Topic Guide: Healthcare Professionals

# Introduction

- 1. Check that participants have read and understood the PIS
  2. Explain arrangements for: consent (cannot withdraw data after the group has finished), recording, anonymity, etc.
  3. Group rules: no right or wrong answers, speaking one at a time, what is said in the room stays in the room etc. (N/A for one-on-one interviews)
  4. Invite participants to expand on responses etc.

# Group introductions:

- 1. Introduce yourself, saying a little about your qualifications, experience, how long you have been in practice/in this current role etc.

# Views and experiences of MSK generally:

- 1. What is your experience of current service provision for this patient population?
  2. What are the strengths?
  3. What are the challenges?
  4. Do these vary for different sub-groups of patients, if so in what ways?

# General views on the use of stratified care

1. What are the advantages and disadvantages of using a stratified care approach with this patient population?
2. What is the relationship between diagnosis and prognosis in determining treatment pathways, and use of stratified care?
3. Does a stratified approach intervention appear more appropriate or inappropriate for particular patients (age, ethnicity, condition specific, etc.) and, if so, under what circumstances?
4. What would you see as the main patient expectations around this model of service delivery?

How can these be addressed?

To what extent would you see patients as having confidence in this model?

1. Are there particular clinician characteristics that impact on the implementation of a stratified approach, i.e. discipline, training, experience etc.?
2. What effects, if any, do differing practice settings have on attitudes to and adoption of stratified care?
3. Are there particular contextual factors that impact on patient outcomes?
4. Are there particular contextual factors that may need to be taken into account for implementation?
5. How do you see this stratified care approach fitting into current and future service provision?

# Close of discussion

- 1. Any other final remarks/additional views.
  2. Check consent is still in place.
  3. Reimbursement of travel expenses etc. (where appropriate).
