# Supplementary material for: Risk-based stratified primary care for common musculoskeletal pain presentations: qualitative findings from the STarT MSK cluster randomised controlled trial
Source: BMC Prim Care. 2022 Dec 16;23:326. doi: 10.1186/s12875-022-01924-3 (PMC9754991; doi:10.1186/s12875-022-01924-3)
Supplement: Supplementary file 2 — Additional file 2. Interview Topic Guide: Patients. [file 12875_2022_1924_MOESM2_ESM.docx]

Interview Topic Guide: Patient

# Introduction

- 1. Check that participant has read and understood the PIS.
  2. Explain arrangements for: consent, recording, anonymity, expenses where appropriate etc.
  3. Invite participants to expand on responses.

# Experience of MSK pain/ previous help-seeking and treatment:

- 1. Ask about previous experiences of MSK pain/ past treatments received etc. (*not in detail, but as context for understanding how patients experienced stratified care*).

# Views on consultation in which stratified care was used:

- 1. Expectations of GP consultation - were these expectations met? If not, what are the reasons for this?
  2. Views about acceptability of the tool questions:
     1. Probe re explanation given by the GP about use/ relevance of tool qu’s – was this sufficient? Did they understand these were being used for treatment matching?
     2. Was there anything important that wasn’t asked?
  3. Views on the treatment options discussed during the consultation:
     1. Any tests ordered/ undertaken.
     2. Decision-making about referrals, e.g. to physiotherapy.
     3. Extent to which they felt involved in decisions about their treatment plan.
     4. Whether they felt they had some degree of choice re treatment options.
     5. If referred to physiotherapy, who initiated this? Did they feel it was suitable/ appropriate for them?
     6. If not referred, was this discussed? Would they have liked this to have been discussed?
  4. Extent to which they felt that their needs were/ weren’t addressed by the GP:
     1. Prompt re degree to which patient felt listened to.
     2. Level of reassurance etc.
  5. What advice did the GP give, and did they followed the advice? If not, why?
  6. Has there been any impact on activities as a result of the consultation? If so, what? If not, any reasons?

# Treatment and management since the GP consultation:

1. Experiences of pain since their consultation, i.e. how has pain progressed, has it resolved etc.?
2. Explore views/ experiences of treatment received (if any) since initial GP consultation:
   1. Prompt re what healthcare professionals they have been to see.
   2. Views on suitability of treatment received.
   3. Waiting time for onward referral - acceptability of this?
   4. Any tests/ investigations undergone?
   5. Expectations of onward treatment, e.g. physiotherapy.
   6. Were these expectations met?
   7. If they received physiotherapy, what advice were they given and did they follow this advice?
   8. Are they satisfied with the care they have received?
   9. Have they been back to see their GP – if so, what prompted the decision to re-consult?
3. If treatment made a difference, explore what patients feel were the key aspects that contributed to this improvement:
   1. Prompt re factors such as engagement in physical activity, confidence/ self-efficacy, therapeutic relationship, reassurance, medication etc.
4. For those with a previous history of treatment for back pain, do they perceive any differences in experiences of treatment/ management from what they have received in the past?

# Close of discussion

- 1. Any other final remarks/additional views.
  2. Check consent is still in place.
  3. Reimbursement of travel expenses etc. (where appropriate).
